# Global Scope of Hospital Pharmacy Practice: A Scoping Review

**DOI:** 10.3390/healthcare8020143

**Published:** 2020-05-25

**Authors:** Aya Ahmed Abousheishaa, Ahmad Hatim Sulaiman, Hasniza Zaman Huri, Syahrir Zaini, Nurul Adha Othman, Zulhilmi bin Aladdin, Ng Chong Guan

**Affiliations:** 1 Department of Psychological Medicine, Faculty of Medicine, University of Malaya, Kuala Lumpur 50603, Malaysia; hatim@um.edu.my (A.H.S.); chong_guan@um.edu.my (N.C.G.); 2 Faculty of Pharmacy, University of Malaya, Kuala Lumpur 50603, Malaysia; hasnizazh@um.edu.my; 3 Department of Pharmacy Practice, Kulliyyah of Pharmacy, International Islamic University Malaysia, Kuantan 25710, Malaysia; syahrirz@iium.edu.my; 4 Pharmacy Department, Hospital Sungai Buloh, Sungai Buloh, Selangor 47000, Malaysia; nuruladha@moh.gov.my; 5 Unit Farmasi, Hospital Bahagia Ulu Kinta, Tanjung Rambutan, Perak 31250, Malaysia; zulhilmi.a@moh.gov.my

**Keywords:** clinical pharmacy, pharmacy practice, clinical services, conventional services, pharmaceutical services, pharmaceutical services, hospital pharmacy, pharmacist, barriers

## Abstract

The pharmacy profession has undergone tremendous changes over the past few decades. Pharmacists’ roles have expanded their boundaries to encompass more patient-centered services. However, the degree to which these roles are practised may vary. This scoping review is aimed at describing the extent and range of the professional pharmacy services offered in hospital pharmacies across different countries and the barriers underlying inappropriate or incomplete implementation of these services. Studies published in the English language between 2015 and 2019 were retrieved from the following databases: PubMed, CINAHL, Scopus, EBSCO Discovery Service, and Web of Science. A thematic analysis across the included studies produced two main themes. “Scope of practice” comprised three subthemes: pharmaceutical care practice, clinical pharmacy practice, and public health services and “Multiple levels of influence” comprised five subthemes: individual, interpersonal, institutional, community, and public policy-related factors. The hospital pharmacy services across countries ranged from traditional drug-centered pharmacy practice to a more progressive, clinically oriented practice. In some countries, there is an apparent inadequacy in the clinical pharmacy services provided compared to other clinical settings. Understanding the current pharmacy practice culture across different health care systems is an essential step towards improving the profession.

## 1. Introduction

The pharmacy profession has undergone tremendous changes over the past few decades that have led to an expansion in the breadth of professional practice. Pharmacists’ roles are no longer limited to the conventional services of drug preparation and distribution; they have extended their boundaries to encompass more patient-centered services to ensure an optimal therapeutic outcome [1,2]. These include educating and counselling patients, promoting health and preventing disease, managing different disease states, and providing specialized clinical recommendations to other healthcare professionals. The widening in the scope of practice accompanied by increased demand for qualified pharmacists was dictated by the elevated occurrence of drug-related morbidity and mortality [3] and the growth in patient needs [4].

A significant challenge related to the analysis of international literature on pharmacy practice is the variability in the definitions of clinical pharmacy and pharmaceutical care. Many countries have endorsed definitions by international organizations and societies, whereas others have developed their own. For this review, we have adopted the American College of Clinical Pharmacists (ACCP) [5] definition of clinical pharmacy as “a health science discipline in which pharmacists provide patient care that optimizes medication therapy and promotes health, wellness, and disease prevention.” The definition further embraces the pharmaceutical care philosophy of blending care, specialized knowledge, experience, and judgment to ensure optimal patient outcomes. Hepler and Strand [6] describe pharmaceutical care as “the responsible provision of drug therapy for the purpose of achieving definite outcomes which improve a patient’s quality of life.” [6] Many countries use it as a working definition; however, its interpretation differs across countries due to the variability in culture, language, and health care systems [7].

The development of coherent regulations by international health care systems has been advocated by the Rio Political Declaration for Health to allow for more practice consistency within and across different settings as well as countries [8]. Several societies for hospital pharmacy were developed in different countries to support the practice of pharmacists in hospital settings. The Basel statements of the International Pharmaceutical Federation’s (FIP) are the first set of harmonious declarations that mirror the future vision of hospital pharmacy practice across the globe. They constitute six main themes: medication procurement, preparation, delivery, administration and monitoring, influences on prescribing, and human resources and training [9]. Pharmacists’ direct contact with patients has been indicated by the Society of Hospital Pharmacists Australia (SHPA) to maintain pharmacists’ roles in medication reconciliation, ward rounds participation, provision of medication information, and monitoring drug therapy [10]. Furthermore, the European Association of Hospital Pharmacists (EAHP) highlighted that the role of pharmacists in hospitals is to “optimize patient outcomes, by collaboratively working within multidisciplinary teams in order to achieve responsible use of medicines” [11]. The efficiency of the drug management process in hospitals is greatly influenced by pharmacy services [12]. Hospital pharmacists are required to provide quality patient care through the implementation of clinical as well as conventional pharmacy services [13]. However, the nature and range of the implemented services may vary across different regions due to disparities in the healthcare structures, public policies, economic resources, culture, and education [4,14].

While there is literature about the pharmacy services offered in different settings and regions, in the context of variability in the implementation of pharmacy services and the lack of literature overviewing these studies, the current level of hospital pharmacy practice across countries remains unclear. Consequently, this scoping review was undertaken to map and synthesize the knowledge available on the extent and range of hospital pharmacy services provided to patients across different countries as well as the factors impeding the appropriate provision of these services. In doing so, gaps in practice can be recognized, paving the path for future research aimed at furthering the pharmacy profession.

## 2. Materials and Methods 

### 2.1. Aim

This review seeks to account for the professional pharmacy services offered in hospital pharmacies across different countries and, in particular, to answer the following research questions: “What are the professional hospital pharmacy services reported in the literature? What are the factors influencing the implementation of these services?” This review aims to recognize the current research studies on the topic and identify the gaps for future research intended to enhance the pharmacist’s role as part of a health care team. The review was guided by the Joanna Briggs Institute (JBI) methodology for JBI scoping reviews [15]. 

### 2.2. Inclusion Criteria

Studies focusing on pharmacists operating within the hospital setting were included in this review. The main concept guiding the review was the type of hospital pharmacy services provided by pharmacists as well as the underlying factors related to the inappropriate or incomplete provision of these services in the context of hospital pharmacies across different countries. Both qualitative and quantitative primary research studies were included in the review, however review articles were excluded.

### 2.3. Search Strategy

A range of databases was included in the search: PubMed, CINAHL, Scopus, EBSCO discovery service, and Web of Science. The search terms used were “clinical pharmacy” or ”pharmacy practice” or “clinical services” or “conventional services” or “pharmaceutical services”; “hospital pharmacy”; “pharmacists” or “pharmacist”; “barriers” or “obstacles” or “challenges” or “difficulties” or “issues” or “problems” or “prevent”; “quality” or “extent”; and “survey” or “questionnaire” or “instrument” or “measure” or “assessment” or “scale”. The review included only full-text English publications between the years 2015 and 2019. Additionally, bibliographies were browsed for supplementary sources.

### 2.4. Selection and Extraction

**Figure 1:**
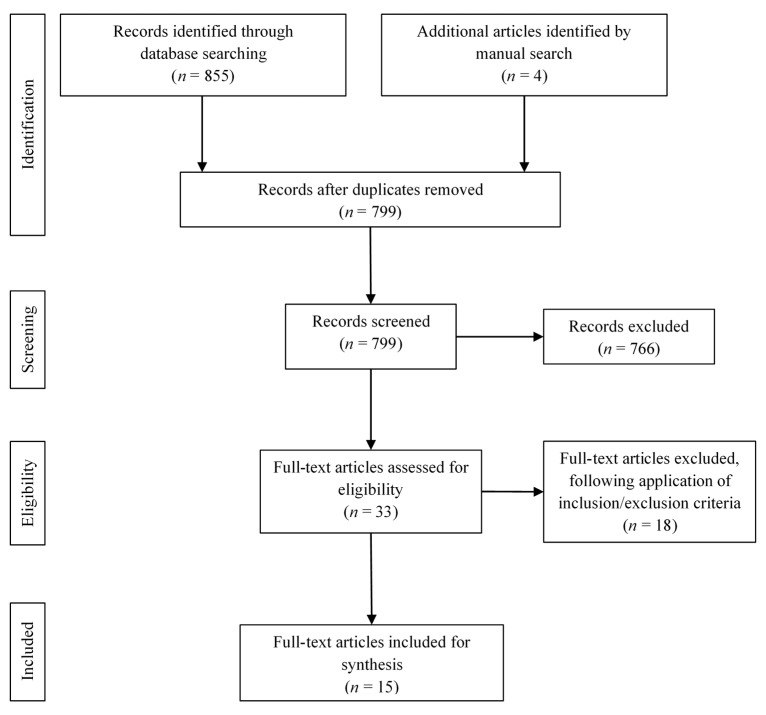
Identification and selection of studies.

A total of 855 abstracts were retrieved from the searched databases. The manual search sourced four additional abstracts. Following the elimination of duplicate results, 799 records remained. Titles and abstracts were checked for apparently relevant studies, leaving 33 for full-text review. Implementing the inclusion criteria resulted in the further elimination of 18 studies, bringing the total to 15 papers for the final review (Figure 1). The following data were extracted and tabulated: author, year of publication, study country, study design and objective, study sample, methodology, core findings, and limitations. The work of Braun and Clark [16] guided the inductive thematic analysis across the studies. This necessitated understanding the outcomes of each study then creating primary codes. Emerging themes were then obtained from these codes and followed by reviewing and refining to generate the final themes.

## 3. Results

This review contains 15 studies, the details of which are abridged in Table 1. Three of the reported studies were conducted in the United States; two in Kuwait; and one study in each of the following countries: Malaysia, Vietnam, Poland, Germany, Qatar, and Switzerland. The remaining studies spanned across countries including Australia and Poland, the United States and Canada, the Pacific Island Countries, and the Western Pacific Region. The design and the focus of the studies differed. Thirteen adopted quantitative design, one was a qualitative exploration, and one was a mixed methods design. Nine studies referred to general hospital pharmacy services, one focused on the Emergency Department (ED), one on Neonatal Intensive Care Unit (NICU), one on oral oncology, one on Parenteral Nutrition (PN), and two on public health services. All the studies sampled subjects including pharmacists, coordinating pharmacists, deputy-heads, or heads of pharmacies, except Langebrake et al., 2015 [17], who studied pharmacist interventions from the German Association of Hospital Pharmacists-Documentation of Pharmacists’ Interventions in the Hospital (ADKA-DokuPIK) database. The key pharmacy services and challenges identified are summarized in Table 2.

### 3.1. Scope of Practice

One of the key emerging themes in this review is the range of hospital pharmacy services offered. There is a substantial variation in the delivery of pharmaceutical services from one setting to another. The disparity is visible in different aspects of the pharmacy practice, including clinical pharmacy, pharmaceutical care, and public health. In particular, areas requiring advanced pharmacy services—like oncology [24], parenteral nutrition [19], emergency care [23], and neonatal intensive care [14]—also reported inconsistent pharmacist roles. 

Authors in three of the reviewed studies portrayed the most common pharmaceutical care services [14,18,19] performed by the majority of the pharmacists. Similarly, nine studies reflected the clinical pharmacy services often offered in the majority of the hospitals [12,17,20,21,22,23,24,25,26,27]. Two studies [28,29] focused on the engagement of hospital pharmacists’ in essential and nonessential public health services. Most of the studies, however, indicated a shift in practice toward nonpatient-oriented pharmacy services. 

#### 3.1.1. Pharmaceutical Care Practice

The practice of pharmaceutical care in Qatar, as portrayed by El Hajj et al., 2016 [18], reflected pharmacists’ involvement in the recognition of drug-related problems and medication counselling. Nevertheless, they seldom formulated patient-specific therapeutic action plans, verified patients’ compliance, followed-up with their physicians, or performed screening activities. Likewise, Poland NICU pharmacists had a more traditional practice, where pharmaceutical care is seldom practised and pharmacists believe they are not part of the NICU team [14]. The area of parenteral nutrition in Kuwait [19] also witnessed predominantly preparation and dispensing rather than direct patient interaction. A limited number of pharmacists had ward-related activities, including participation in patient assessment and monitoring as well as the design of total parenteral nutrition regimens. The pharmaceutical care practice in NICU in Australia [14], on the other hand, was more progressive, involving the provision of drug recommendations, resolution of drug-related problems, and medication review encompassing both clinical and dispensing services. 

#### 3.1.2. Clinical Pharmacy Practice

In Polish hospitals [12], the clinical pharmacy services were described as not highly developed; most of the pharmacists were engaged in the extemporaneous preparation of sterile and non-sterile dosage forms, inpatient medication supply, and drug and therapeutics committees. Involvement in clinical trials and the preparation of parenteral nutrition and cytotoxic medications was mainly confined to hospitals located in large cities and employing a higher proportion of professionals. Patient-centered pharmacy services were found to be very limited; only 4 out of 100 pharmacists were involved in hospital ward rounds. Direct patient contact was initiated by a small number of pharmacists, as the majority did not regard themselves as clinical pharmacists and collaborated with other health care professionals on administrative rather than clinical matters. Congruently, in Kuwait [20], only a small fraction of the pharmacists’ time was dedicated to clinical pharmacy services. Furthermore, pharmacists expressed reluctance towards the future provision of these services. In Vietnam [21], the protocol governing adverse drug reaction monitoring involved reporting by physicians and nurses, while pharmacists acted as a liaison with the authorities only. Furthermore, the scope of information services was also limited to the collection and dissemination of information, thereby creating a gap between the number, quality, and impact of these services. Patient-oriented pharmacy services reported by almost half of the surveyed hospitals involved the attainment of accurate patient medication history and identification of drug-related problems. 

Different regions of Switzerland offered heterogenous pharmacy services [22], with 84% of the surveyed hospitals having no structured clinical pharmacy services. Multidisciplinary ward rounds, therapeutic recommendations in specific wards, and the provision of drug information to other health care professionals were the main clinical pharmacy services offered. Similarly, the nature of pharmacy services offered in the United States Veteran Affairs medical centers [23], varied considerably from one center to another. The services provided included medication reconciliation, pharmacotherapeutic recommendations, patient counselling and education, reporting adverse drug reactions, ensuring formulary adherence, obtaining drug history, and attending preceptor roles. In the field of oncology [24], less than half of the surveyed pharmacists were involved in medication therapy management (MTM), oral chemotherapy, and collaborative practice agreements (CPA). Pharmacists provided patient education, assisted patients with financial matters, altered medications, requested and evaluated lab tests, and formulated therapeutic plans.

Then again the Pacific Island Countries [25] depicted the active involvement of hospital pharmacists in enhancing patient care despite their limited number. The majority of the studied hospitals reported having pharmacy and therapeutics committees to manage the medicine formulary system. Similarly, Jonathan Penm et al., 2015 [26] reported that the majority of the hospitals surveyed in the western pacific region offered some form of clinical pharmacy services as per the International Pharmaceutical Federation (FIP) statements. Ward participation included obtaining comprehensive medication history, medication review, and counselling patients on discharge. In Germany, the day to day clinical pharmacy interventions [17] had an implementation rate of 85%. The services were mainly related to general rather than specialized areas of health care, including the surgical ward, internal medicine, anesthesiology, intermediate care unit, and intensive care unit. The interventions included suspending drugs, changing drug dose, or changing drugs, and were mainly involving elderly patients. 

#### 3.1.3. Public Health Services 

In Iowa and North Dakota [28], pharmacists generally played a role in disease state management, medication therapy management, care transition from inpatient to outpatient, prescription medication take-back, smoking cessation, and vaccination. Essential public health services—like the enforcement of health protection related laws and regulations, patient education on health-related issues, and participation in training outside the scope of continuing education requirements—were witnessed in Iowa, North Dakota, and Manitoba [29]. However, in Vietnam hospitals, pharmacists were not directly involved in health protection laws implementation [21]. Pharmacists were least involved in needs assessments for the identification of community health risks, community partnership for the recognition and resolution of problems, and advocacy towards policy amendments [28,29].

### 3.2. Multiple Levels of Influence

#### 3.2.1. Individual Factors

Across the studies, individual aspects played a role in the prevalence and scope of hospital pharmacy services. Trinh et al., 2018 [21] highlighted that pharmacists lacked the necessary skills required for the successful operationalization of the medication review process, thereby limiting its effectiveness. Similarly, several studies [18,19,20,27] emphasized the importance of pharmacists’ education to allow for the expansion of their practice scope. 

Pharmacists’ attitude can potentially impact their behavior [30]. Several studies have reported positive attitudes towards different aspects of hospital pharmacy services, including medication therapy management [27], rational drug use [12], pharmaceutical care [18], collaborative patient care [19], and contribution to pharmacotherapeutic decisions [14]. Then again, some pharmacists were less enthusiastic about pharmaceutical care [18], while others lacked interest in clinical pharmacy services [12]. 

Lack of confidence was reported as a challenge hampering the provision of public health services [28]. On the other hand, the acceptance of professional responsibility towards prescribed medication motivated pharmacists to embark on the provision of clinical pharmacy services [25].

#### 3.2.2. Interpersonal Factors

Pharmacists’ relationships with other health care professionals have been found to influence the expansion of hospital pharmacy services. Pharmacists reported that a good rapport with other healthcare team members motivated them to enhance clinical pharmacy practice [25]. Furthermore, intense competition between pharmacists and dispensing physicians was found to hamper interdisciplinary collaboration, thereby leading to diminished clinical pharmacy services [22]. Likewise, the lack of collaborative care was reported as a challenge to the enhancement of practice [18,19].

#### 3.2.3. Institutional Factors

Several institutional related issues challenge the expansion of the pharmacists’ roles. The scarcity of staff or human resources has been consistently highlighted in nine of the reviewed studies [12,18,19,20,21,22,26,28]. Lack of time [12,18,24,27,28,29] and work overload [12] were also reported as potential barriers. From the financial perspective, reimbursement models or plans were aspects highlighted for future the expansion and implementation of hospital pharmacy services [18,24,28,29]. Pharmacists also indicated the need for accessibility to references, current practice guidelines [19,27], and patient medical records [18] to facilitate their practice. Furthermore, the lack of managerial support [29] and the lack of mentors [28] were perceived as factors hindering the implementation of public health services. Other institution-related factors extracted from this review were poor hospital infrastructures [21] and high implementation cost [27].

#### 3.2.4. Community Factors

A few studies reported some community-related factors that influenced the prevalence of hospital pharmacy services. Patient demand and expectation were considered essential factors in the expansion of pharmacist roles [26,28]. Physicians’ expectations motivated pharmacists in western pacific countries towards practicing clinical pharmacy [26]. Furthermore, their acceptance of the pharmacists’ recommendations had an impact on the pharmacists’ contribution to ward rounds [21].

#### 3.2.5. Public Policy

Jonathan Penm et al., 2015 [21] indicated that government support increased the likelihood of the implementation of patient-oriented pharmacy services in the Western Pacific Region. Likewise, the lack of official policies [26] and standardized protocol [18] hampered the expansion and implementation of clinical pharmacy in Kuwait.

## 4. Discussion

Despite there being a large body of literature on the subject of hospital pharmacy services, most of it is concerned with specific countries or regions. Pharmacy services have increased in variety and complexity to accommodate different health care systems and a wide range of medical conditions across countries. This shift in the profession increases the need for clinically oriented practice and collaboration with other healthcare professionals. This scoping review is the first overview and synthesis of literature concerned with research describing hospital pharmacy practice and its challenges across different geographical regions. It contributes, therefore, to the progress of pharmacist-led services on an international level through the recognition of gaps in practice.

Our findings indicate variability in the hospital pharmacy services delivered to patients. It highlights the significant inconsistency in pharmaceutical care, clinical pharmacy, and public health services across several countries. Some healthcare systems are navigated towards traditional pharmacy services mainly centered at medication supply, whereas others have laid structures to accommodate clinical services. These variances are a result of multiple levels of influence, involving individual, interpersonal, institutional, community, and public policy-related factors. However, most hospitals strived towards progressing practice, often through increased involvement with direct patient care. Comparing the current health care practice against the envisioned future healthcare state contributes to advancing the profession through gap analysis [31]. It is desirable that countries with poor practice standards benchmark countries with better practice standards. To benchmark pharmacy services, a three-tier system permits comparison against recommended practice standards and fellow pharmacists and against oneself over time [32]. 

The World Health Organization instigated the following roles for pharmacists irrespective of the practice setting; caregiver, decision-maker, communicator, manager, life-long learner, teacher, and researcher [33]. However, there are arguments against universal standards of practice, especially considering the variability in pharmacists’ training and the broad scope of services and responsibilities [34]. Nevertheless, countries should endeavor to provide uniform progressive pharmacy services to warrant patients equal healthcare opportunities. The World Health Organization recognizes global health equity as a priority across different healthcare systems, and it encourages consistent health-related services within and between hospitals [35]. The standardization of health care services can increase efficiency, mitigate risk, and reduce medical costs; it contributes to homogenous patient outcomes [36]. The World Health Organization acknowledges that the standardization of best health care practices is a prime challenge in the enhancement of patient safety. Hence, it advocates the development and implementation of standard practice tools like the WHO High 5′s Project that could be adapted for hospitals both nationally and internationally [36]. The benefits of standardized care extend beyond patients to policymakers through facilitating the benchmarking of healthcare services between hospitals, laying the groundwork for the assessment of health care practices, and allowing the comparison of patient outcomes [36]. The findings of this review emphasize the importance of the global operationalization of enhanced pharmacy services. 

This scoping review, despite its contribution to the knowledge of pharmacy practice, has some limitations that need to be acknowledged. It explored the hospital pharmacy services across different countries and factors impeding its appropriate implementation. There is a potential lack of specificity due to the variety of countries represented by the research studies contained within this review. Furthermore, there might be potential bias from the studies included due to the lack of quality appraisal, which is not a constituent of the scoping review methodology. Although the review is broad, some relevant reports may have been overlooked, since only English language studies were included. Additionally, some of the studies included in the review were qualitative, the findings of which may not be generalized due to participant-specific subjectivity within a particular context and researcher-related variability in data analysis and interpretation. Nevertheless, this review highlighted the disparity in pharmacy-related health care services within and between different hospitals across countries, indicating the need for future research on harmonizing the practice. 

## 5. Conclusions

The hospital pharmacy services across countries ranged from traditional drug-centered pharmacy practice to a more progressive, clinically oriented pharmacy practice. In some countries, there is an apparent inadequacy in the clinical pharmacy services provided compared to other clinical settings. The practice was affected by multiple levels of influence, including individual, interpersonal, institutional, community, and public policy-related factors. Researchers in the field of hospital pharmacy practice are encouraged to publish their work in international journals. Understanding the current pharmacy practice culture across different health care systems is an essential step towards improving the profession. The standardization of progressive pharmacy practices involving direct patient care contributes to enhanced patient safety. Further research is required to develop and operationalize global pharmacy practice guidelines and policies in all specialties. 

## Figures and Tables

**Table 1:** Papers exploring hospital pharmacy services.

Author, Year	Country	Design and Objective	Sample (*n*)	Methodology	Core Findings	Limitations
El Hajj et al., 2016 [18]	Qatar	Quantitative Describe the practice of PC and explore the challenges to its implementation and evaluate pharmacists’ level of understanding of PC and their attitudes towards the practice.	Pharmacists (274)	Cross-sectional survey.	The majority of the pharmacists had an accurate understanding of the aim and their role in PC. However, less than half knew the role of the patient in PC. Not much time was spent on PC activities. The main challenges reported included a lack of accessibility to patient medical information, staff, and time.	Low response rate, non-respondent bias, social desirability bias.
Krzyzaniak et al., 2018 [14]	Australia, Poland	Quantitative Describe and compare the pharmacy services performed in NICUs in Australia versus Poland.	Pharmacists (52)	Cross-sectional online survey.	A higher percentage of clinical services were offered in Australia compared to Poland, including drug recommendations, drug therapy problems interventions, and patient medication chart review.	The sample may not be representative of both countries.
Katoue & Al-Taweel, 2016 [19]	Kuwait	Qualitative Explore the therapeutic role of pharmacists in PN, their information sources, their thoughts on NSTs, challenges to PC practice, and opinions on its enhancement.	TPN pharmacists (7)	Semi-structured interviews.	Pharmacists were mainly involved with technical tasks with minimal patient care. Despite preferring to work within NSTs, no hospital had any functioning teams. The reported challenges included a lack of reliable information sources, lack of SOPs, staff, and time limitations, as well as poor communication.	Small sample size, social desirability bias.
Pawłowska et al., 2016 [12]	Poland	Quantitative Explore the implementation of both clinical and traditional pharmacy practice in Polish general hospitals.	Head pharmacists (166)	Cross-sectional survey	Most participants were involved in drug procurement and circulation, compounding, monitoring ADR, and drug management services. Only 7% were involved with patients and 4% did ward rounds. The main challenge reported was the lack of precise hospital pharmacy practice legal regulations.	Potential misinterpretation of the survey questions.
Lemay et al., 2018 [20]	Kuwait	Quantitative Document existing CPSs, identify challenges to their implementation, and evaluate pharmacists’ perceptions on the future CPSs across public hospitals.	Pharmacists (166), Physicians (284)	Cross-sectional survey.	More than 50% of the pharmacists provided CPSs mainly related to providing education and drug information. The majority were not sure about the future extension of the breadth of their services. A total of 97% of physicians were positive about the clinical role of the pharmacist. Major reported challenges included a lack of policy, time, and clinical skills.	Limited to governmental hospitals.
Trinh et al., 2018 [21]	Vietnam	Mixed methods Explore the CPSs as well as the facilitators and challenges in implementing them in Hanoi hospitals.	Quantitative: Head/deputy head pharmacists (39)Qualitative: Pharmacists (20)	Cross-sectional online survey and in-depth interviews.	The majority of the CPSs were nonpatient-specific, including providing drug information, ADR reporting, and monitoring of drug usage. Reported barriers included a dearth of workforce and competent clinical pharmacists.	Small sample for the interviews; the study involved one province only.
Messerli et al., 2016 [22]	Switzerland	Quantitative To map the provision of CPSs and to discuss their development process.	Pharmacists (47)	Cross-sectional survey.	The majority of the hospitals offered CPS. A total of 73% involved weekly multidisciplinary ward rounds and 9.1% performed medication reconciliation daily.	Services reported based on local needs.
Owenby et al., 2015 [23]	United States	Quantitative Determine the prevalence and the types of pharmacy services and the attitude towards future pharmacy services in Veteran Affairs Emergency Departments.	Pharmacists (33)	Cross-sectional online survey.	The core pharmacy services implemented included medication reconciliation, educating/counseling patients, recommending pharmacotherapy, educating healthcare professionals, precepting activities, reporting ADR, and maintaining compliance with the formulary.	Low response rate of 21.6% and possible selection bias.
Holle et al., 2017 [24]	United States	Quantitative Identify pharmacy services in the field of oral chemotherapy programs, MTM, and CPAs.	ACCP and Hematology/Oncology PRN Pharmacists (81)	Cross-sectional survey.	A total of 35% of the respondents provided MTM services, with a small proportion performing quality assurance evaluations. The core CPA activities included medication adjustment, requisition, interpretation, and monitoring lab evaluations, development of therapeutic plans, and patient education.	Low response rate of 10%, restricted to the members of the ACCP and the Hematology/Oncology PRN.
J. Penm et al., 2015 [25]	Pacific Island countries	Quantitative Explore hospital pharmacy services as well as hospital pharmacists’ effect on medication prescriptions and quality use of medicines.	Head pharmacists (55)	Cross-sectional online survey.	More than half of the hospitals had CPSs with an average of two pharmacists onboard. The majority had a formulary, as well as a Pharmacy and Therapeutics Committee. Participants also believed they had a good relationship with other HCP and good communication skills as well as took professional responsibility for the prescribed medications.	Low response rate and missing data.
Jonathan Penm et al., 2015 [26]	Western Pacific region	Quantitative Explore the implementation of CPSs that influence prescribing and the facilitators and challenges of their practice.	Head pharmacists (726)	Cross-sectional online survey	A total of 90.6% of the hospitals reported providing CPSs, with an average of 28% pharmacists performing regular medical rounds. A total of 30% of the inpatients receive medication reconciliation and discharge counselling. Facilitating factors include government sustenance, physician, and patient prospects.	Selection bias, non-response bias, low response rate of 29%, item non-response.
Langebrake et al., 2015 [17]	Germany	Quantitative Describe and evaluate the extent of pharmacists’ interventions in the ADKA-DokuPIK database.	Pharmacist interventions (27610)	Retrospective descriptive analysis.	The rate of implementation of the PIs was 85.5%. It mainly involved dose change, drug change, and drug suspension.	No successive documentation of all pharmacists’ interventions.
Al-Tameemi & Sarriff, 2019 [27]	Malaysia	Quantitative Assess the KAP of pharmacists at Hospital Pulau, Pinang on MTM services, identify the barriers towards the future provision of such services.	Pharmacists (93)	Cross-sectional survey.	Most of the respondents had a high level of knowledge of MTM. All agreed it could enhance the quality of health care and the majority were keen on providing such services. The potential barriers included lack of training (88.2%), budget (51.6%), and time (46.2%).	Small study only one hospital involved.
Scott et al., 2016 [28]	United States	Quantitative Assess the frequency of public health and essential services delivery and barriers to their expansion among rural and urban Iowa and North Dakota pharmacists.	Pharmacists (602)	Cross-sectional online survey.	Pharmacists in rural areas reported a higher frequency of delivery of public and essential services including MTM, immunizations, tobacco counselling, drug disposal programs, evaluation of pharmacy service provision, partnership with the community on health problems, and assessment of community health risks.	Low response rate; a small study involving two states.
Strand et al., 2017 [29]	United States, Canada	Quantitative Determine and compare pharmacists’ views of their involvement in the 10 essential public health services in Iowa, North Dakota, and Manitoba.	Pharmacists (649)	Cross-sectional online survey.	The main practised services included the enforcement of health and safety protection laws and regulations, public counselling on health issues, and participation in training.	Recall bias, non-response bias, low response rate.

HCP: Health Care Professional, KAP: Knowledge Attitude and Practice, MTM: Medication Therapy Management; ADR: Adverse Drug Reaction, CPS: Clinical Pharmacy Services, CPA: Collaborative Practice Agreement, ACCP: American College of Clinical Practice, PRN: Practice and Research Network, PC: Pharmaceutical Care; NICU: Neonatal Intensive Care Units; PN: Parenteral Nutrition; NST: Nutrition Support Team, SOP: Standard Operating Procedure; TPN: Total Parenteral Nutrition.

**Table 2:** Key emerging themes.

Main Themes	Specific Aspects	Sources	Sample Quotation
Scope of practice	Pharmaceutical care	[14,18,19]	“We only receive the TPN orders and compound the TPN bags. We don’t see the patients” [15]. “Nursing staff have become reliant on medication guidelines and are hesitant to work outside of these guidelines without pharmacy involvement” [11].
	Clinical pharmacy	[12,17,20,21,22,23,24,25,26]	“Clinical pharmacists only pool the needs of clinical wards then submit it to PTC. [They] are not involved in process to add or remove medicines in the hospital formulary” [21]. “Collaboration is only associated with the preparation of drugs for the ward, formulations for individual patients such as powders, feeding bags or antibiotics… contact with doctors is very limited. The most common contact is with the NUM” [11]. “The common tasks of clinical pharmacists in clinical wards are checking the indications and contraindications, evaluating the drug choice, dosage…discussing the intervention with doctors” [21].
	Public health	[21,28,29]	“Clinical pharmacists receive ADR reports which was reported directly from clinical wards or [clinical pharmacists] check ADR logbooks in clinical wards during weekly hospital investigations. Next [clinical pharmacists] enquire about missed information and write the report, then send to the National Drug Information and ADR Monitoring Centre” [21].
Multiple levels of influence	Individual factors	[12,14,18,19,20,21,25,27,28,30]	“I would like to provide pharmaceutical care but simply I do not know where or how to start, and I am not comfortable with taking risks associated with assuming responsibility for the treatment outcomes of patient” [17]. “Our background knowledge regarding TPN from our undergraduate study is limited and the type of work we are involved in is critical. We need more training” [15].
	Interpersonal factors	[18,19,22,25]	“The physicians believe that PN therapy is their own responsibility. They take over all the decisions related to TPN [15].” “Great multidisciplinary team-work. The NICU pharmacist is an integral part of the team. Effective rapport and communication between medical staff, nursing staff and pharmacist. Regular consultation for pharmacist input during medical rounds, and throughoutthe day” [11]. “Doctors haven’t been familiar with clinical pharmacists’ interventions yet. HCPs at clinical wards are still afraid [of pharmacists] because for a long time ago, pharmacists used to come to clinical wards to check the medication boxes. So [we] really want to change the attitude of other HCPs” [21].
	Institutional factors	[12,18,19,20,21,22,24,26,27,28,29]	“I think the absence of this team is due to organizational issues, e.g., lack of guidelines to develop NSTs at hospitals. In addition, there may be insufficient staff to establish NST” [15].
	Community factors	[21,26,28]	“Clinical case discussions are regular; sometimes take place in grand rounds at our hospital. Identified medication errors are more likely accepted by physicians when the director and head of the administration department were there…” [21].
	Public policy	[19,20,26]	“Unfortunately, we don’t have any continuing medical education (CME) activities related to TPN in Kuwait” [15]. “We don’t have a standard reference for our work. Each hospital has its own TPN protocol which is different from one hospital to another. This can create communication problems among the hospitals” [15].

NICU: Neonatal Intensive Care Units; PN: Parenteral Nutrition; NST: Nutrition Support Team; TPN: Total Parenteral Nutrition.
